# Fluoropyrimidine Toxicities and DPYD Genotyping: A Clinical Case

**DOI:** 10.7759/cureus.63814

**Published:** 2024-07-04

**Authors:** Maurício Peixoto, Diana Alves, Altina Lopes, Luísa Queiróz, Marta Almeida

**Affiliations:** 1 Medical Oncology, Unidade Local de Saúde de Braga, Braga, PRT; 2 Human Genetics-Neonatal Screening, Metabolism, and Genetics Unit, Instituto Nacional de Saúde Doutor Ricardo Jorge, Porto, PRT

**Keywords:** dpyd, case report, toxicity, dihydropyrimidine dehydrogenase, fluoropyrimidines

## Abstract

Fluoropyrimidines are metabolized mainly by the enzyme dihydropyrimidine dehydrogenase (DPD). Some variants in the coding gene, *DPYD*, have already been associated with increased toxicity, and their testing is recommended in patients proposed for treatment with these drugs.

In this clinical case, a patient without the four variants recommended for testing showed toxicity from the second cycle of capecitabine onwards, requiring hospitalization in the third cycle. Sequencing of the *DPYD* gene showed the presence of three heterozygous variants which the laboratory interpreted as deleterious to the expression or function of DPD. In the absence of phenotypic testing, the patient stopped treatment, which is also the usual procedure in the case of compound heterozygosity of the variants usually tested.

The literature review shows data for and against the role of each variant found in fluoropyrimidine toxicity, but there may be more concrete data in the study of haplotypes with multiple variants and the need to research alterations in other genes to better understand their relationship with toxicity.

## Introduction

Fluoropyrimidines, such as 5-fluorouracil (5-FU) and capecitabine, are cytotoxic drugs used to treat various solid tumors, particularly gastrointestinal, breast, and head and neck cancers. Their mechanism of action involves inhibiting the enzyme thymidylate synthase and the incorporation of the drug's metabolites into RNA and DNA. More than 80% of these drugs are metabolized in the liver by the enzyme dihydropyrimidine dehydrogenase (DPD), encoded by the *DPYD* gene. Several polymorphisms of this gene are known, some of which result in alterations in the expression and/or function of DPD. There are guidelines for researching four variants of the *DPYD* gene (c.1905G>A, c.1679T>G, c.2846A>T, and c.1129-5923C>G) because they are relatively common and have clinical relevance for enzyme function. These variants can lead to increased toxicity in patients treated with fluoropyrimidines, indicating the need for dose reduction or avoidance of this drug family [1,2]. However, there are other polymorphisms with conflicting reports on their effect on the toxicity of these drugs [3].

## Case presentation

**Figure 1:**
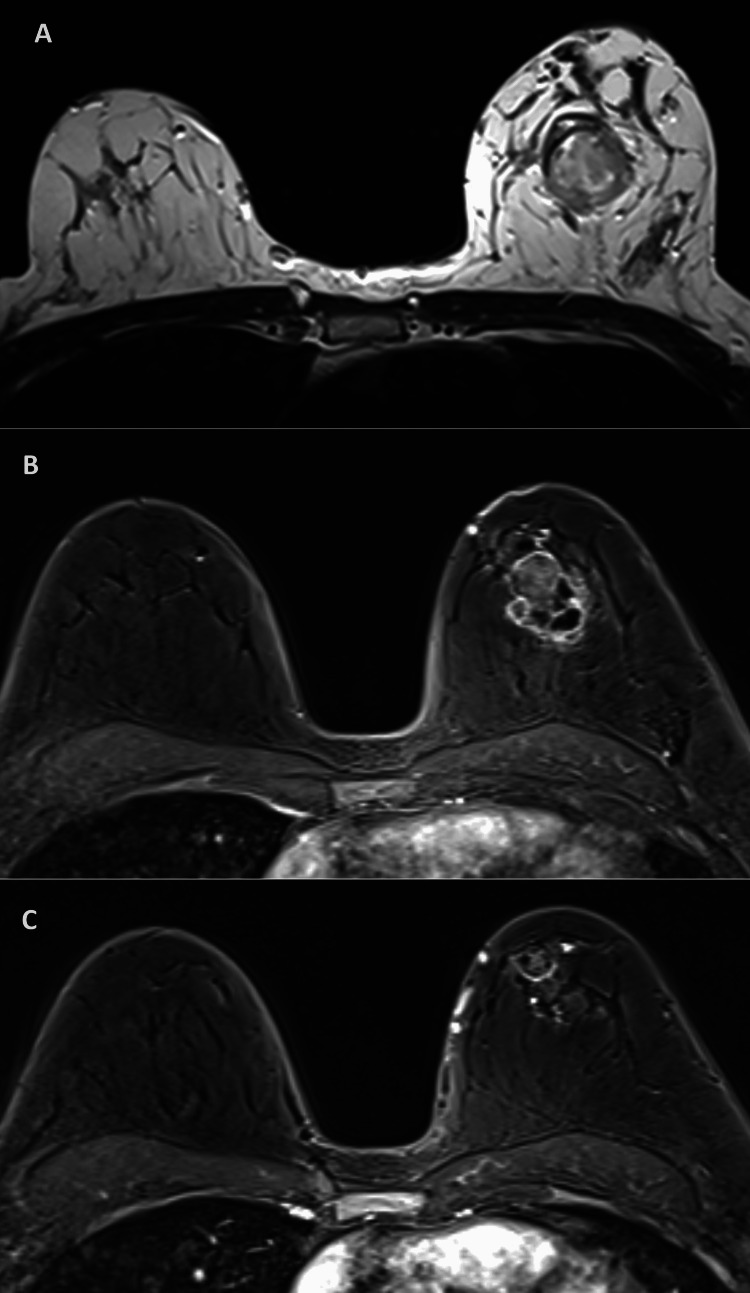
Breast MRI (A) Before neoadjuvant chemotherapy. (B) After neoadjuvant chemotherapy: some necrosis is visible indicating a response to treatment, but also present anterior and medial proliferation. (C) After neoadjuvant chemotherapy: new lesion located above the original tumor indicating progression

A 52-year-old woman, with no relevant past medical history, was diagnosed with triple-negative non-specific carcinoma (NST) of the left breast, clinically staged as cT2 N0 M0. She underwent neoadjuvant chemotherapy (12 cycles of weekly paclitaxel at 80 mg/m^2^ and four cycles of carboplatin at AUC6 every three weeks followed by four cycles of doxorubicin at 60 mg/m^2^ and cyclophosphamide 600 mg/m^2^ every two weeks according to the department protocol) having clinical progression confirmed by breast MRI (Figure 1). She underwent total mastectomy and sentinel node biopsy; histology of the specimen showed invasive carcinoma NST ypT2N0sn (three sentinel nodes) without the expression of estrogen receptors (ER), with 10-15% of progesterone receptors (PR) and absence of HER2 expression. The patient was offered adjuvant therapy with capecitabine (1000 mg/m² twice a day for the first 14 days of each 21-day cycle), having been tested for the four genetic variants of the *DPYD* gene recommended by the European Society for Medical Oncology (ESMO) without any of them being detected. After the first cycle, the patient complained of dyspepsia, which resolved with medical treatment. In the second cycle, she started grade (G) 2 diarrhea, cramps, nausea, and vomiting. The patient was medicated with butylscopolamine, loperamide, probiotics, metoclopramide, and ondansetron. Despite the therapy initiated, the patient's symptoms worsened, presenting uncontrollable vomiting, G3 diarrhea, cramps, and eructation, with the need for hospitalization for symptomatic control.

The patient remained hemodynamically stable and presented only abdominal pain on palpation in the lower quadrants on physical examination. She began intravenous antibiotic therapy with ciprofloxacin after collecting fecal samples for microbiological and parasitological analysis and *Clostridium* testing and stopped her oral diet. During hospitalization, the patient had G3 neutropenia, G2 anemia, G1 thrombocytopenia, and G1 alanine aminotransferase elevation and maintained G1 diarrhea.

Given the onset of the condition after the second cycle and the worsening with the start of the third cycle of capecitabine, the search for other genetic variants of the DPD gene that could justify the probable toxicity observed was discussed. During hospitalization, the patient progressively improved with supportive measures and antibiotic therapy. Stool tests did not reveal any causative agent, and the patient also underwent an abdominal and pelvic CT scan which only showed ileitis, possibly secondary to chemotherapy or infection, which justified adding metronidazole to the ongoing antibiotic therapy. The patient improved progressively, and by the eighth day of hospitalization, she had no complaints and was discharged home, still awaiting the study of the *DPYD* gene variants to discuss the reintroduction of the drug.

The study of the gene, conducted at Instituto Nacional de Saúde Doutor Ricardo Jorge, utilized next-generation sequencing (NGS) and included the sequencing of all the exons and respective flanking intronic regions of the *DPYD* gene. It was performed. The results showed the presence of three heterozygous variants, namely, c.85T>C, c.496A>G, and c.2194G>A. The laboratory, following the Human Gene Mutation Database, interpreted the c.85T>C and c.496A>G polymorphisms as associated with DPD deficit and the c.2194G>A as a variant that reduced the enzyme's activity. In view of the toxicity observed, the laboratory's interpretation, and the fact that the patient was heterozygous for three variants of the *DPYD* gene and that it was impossible to test the function of DPD at our center, we decided to suspend adjuvant treatment with capecitabine.

## Discussion

We present a case of significant toxicity during adjuvant capecitabine treatment in a woman with breast cancer. This toxicity led to the sequencing of the *DPYD* gene to look for polymorphisms that could be involved in this toxicity. The variants c.85T>C, c.496A>G, and c.2194G>A were found in heterozygosity. These variants have all been reported previously.

The c.85T>C variant, despite the laboratory's interpretation, has often been considered harmless or as an enzyme function enhancer [3]. Božina et al. showed that there were no differences in toxicity between c.85 genotypes [4], Hamzic et al. showed that the haplotype with only the c.85 T>C variant is associated with increased plasma dihydrouracil/uracil (HU2/U) ratios [5], which may indicate greater enzymatic activity, and Medwid et al. [6] also found no association with severe toxicity to fluoropyrimidines and the haplotype with the isolated c.85T>C variant. However, in this study, haplotypes combining the c.85T>C and c.496A>G variants were associated with severe toxicity to fluoropyrimidines, whereas the c.496A>G variant alone did not show this association [6]. Hamzic et al. also showed that haplotypes containing the c.496A>G variant alone or accompanied by c.85T>C were associated with a decrease in the HU2/H ratio [5]. Gross et al. had already shown in 2008 that there was an association between the c.496A>G variant and greater toxicity in breast cancer patients [7]. Božina et al. had shown a significant importance of the c.496A>G variant in increasing the risk of toxicity [4], which had also been affirmed by Falvella et al. in 2015 in patients with colorectal cancer [8]. In 2018, Del Re et al. showed only a non-statistically significant trend towards increased toxicity with the c.496A>G variant, but a significant association with the c.2194G>A variant, which was also found in this case [9]. This variant, which the laboratory interpreted as reducing enzyme activity, was associated with a higher risk of neutropenia and a shorter time to adverse effects in the study by Iachetta et al. [10]. Božina et al. had also shown an increased risk of toxicity associated with this variant, but not as much as c.496A>G [4]. This association was also shown in the PETACC-8 study [11]. Kim et al. conducted a meta-analysis in which they reported an association between the c.2194G>A variant and an increased risk of toxicity [12]. However, in the analysis of patients in the QUASAR II study by Rosmarin et al., none of the three variants found in this case were associated with an increased risk of toxicity [13]. Lešnjaković et al. carried out a review on clinically relevant variants in which they discuss the role of these three variants, showing the body of literature already published that is for and against the role of these isolated variants, concluding that more studies are needed on the role of *DPYD* variants in fluoropyrimidine toxicity, including the importance of haplotypes that bring together more than one variant [3]. Gentile et al. showed that the B7 haplotype, which combines the three variants found in this case, leads to a reduction of over 50% in the rate of 5-FU degradation, potentially indicating a stronger association with toxicity [14]. In addition to the haplotypes with variant conjugation, there are other genes involved in fluoropyrimidines metabolism that may harbor polymorphisms that could influence drug toxicity within this drug class [15]. Investigating the impact of both the haplotypes and these additional genes could provide valuable insights into predicting fluoropyrimidine toxicity.

## Conclusions

In this clinical case, the patient presented exuberant side effects requiring hospitalization even though the recommended study of *DPYD* variants was negative. Given the severity of the symptoms, it became imperative to search for less frequent variants. Although the literature shows divergent data regarding the variants found, in this case, given the patient's clinical presentation, it was assumed that the combination of the three variants was responsible for the toxicity, leading to the suspension of treatment. This clinical case reflects the importance of searching for less frequent variants in cases of severe toxicity.
